# Design and UV-curable behaviour of boron based reactive diluent for epoxy acrylate oligomer used for flame retardant wood coating

**DOI:** 10.1080/15685551.2016.1231029

**Published:** 2016-10-13

**Authors:** Sachin U. Chambhare, Gunawant P. Lokhande, Ramanand N. Jagtap

**Affiliations:** ^a^ Department of Polymer and Surface Engineering, Institute of Chemical Technology, Mumbai, India

**Keywords:** Boron based reactive flame retardant, UV-curable wood coating, limiting oxygen index (LOI) UL-94 burning test

## Abstract

Difunctional boron-containing reactive flame retardant for UV-curable epoxy acrylate oligomer was synthesized from phenyl boronic acid and glycidyl methacrylate. The synthesized reactive diluent was utilized to formulate ultraviolet (UV)-curable wood coatings. The weight fractions of reactive diluent in coatings formulation was varied from 5 to 25 wt % with constant photoinitiator concentration. The molecular structure of reactive flame retardant was confirmed by Fourier-transform infrared, Nuclear magnetic resonance (NMR) and ^11^B NMR spectral analysis. Further, the efficacy of flame retardant behaviour of coatings was evaluated using limiting oxygen index and UL-94 vertical burning test. Thermal stability of cured coatings films were estimated from thermogravimetric and differential scanning calorimetry analysis. The effects of varying concentration of reactive diluent on the viscosity of coatings formulation along with optical, mechanical and chemical resistance properties of coatings were also evaluated. The coatings gel content, water absorption behaviour, contact angle analysis and stain resistance were also studied.

## 1. Introduction

With the increasing awareness regarding the health, fire safety and environmental regulations, demands for reduction of the fire hazard posed by highly combustible materials such as wood, plastics, textiles, etc., have gained significant importance in recent past. Therefore, to avoid the fire hazards, design of environmentally friendly flame retardant is critical which might be able to retard the ignition of these materials or decrease flame spread, thereby obviating fire hazards and loss of life and destruction of property.[1]

Amongst the various materials, wood is an organic natural composite of cellulose fibres even today widely used for constructions, decorative furniture’s etc. due to its flexibility in designing and ease of processability. It is suitable not only as a finish material, bringing warmth and natural beauty to the interior but also as a structural material, offering a cost-effective way to meet building code requirements for safety and performance.

However, it is highly vulnerable to fire accidents as its backbone is made up of carbonaceous cellulose composite. Therefore, to improve the fireproof capability of wood (or wood-based material) becomes one of the important challenge, which has attracted substantial attentions of the researchers and force them to develop suitable and eco-friendly flame retardant coating.

Especially, in the field of UV-curing industries epoxy and epoxy derivatives have been widely used in different industrial sectors like coatings, structural adhesives, advanced composite materials and for encapsulating electronic microchips in electronic devices due to their good chemical and physical properties which is the outcome of characteristics structural feature of epoxy backbone. However, their wider applications in different sectors are restricted by their high flammability as it is it being highly enriched with carbonaceous chemical moiety. The flammability properties of epoxy resins can be improved by additive and/or covalent approach. Traditionally, organic bromine compounds with antimony oxide, ammonium polyphosphate, metal hydroxides and red phosphorus were widely used as a flame retardant additive for epoxy resins.[2,3]

Traditionally, organo-halogenated compounds have been most widely used as flame retardant materials either as reactive co-reactant or additives, particularly in the composite or in electronic equipment. However, halogenated flame retardant and their derivatives were suffered from various disadvantages, such as upon combustion, they generate corrosive and obscuring smoke along with highly corrosive fumes of halo acids that corrode the metal components. In addition, they also produce extremely carcinogenic substances like dibenzodioxins and dibenzofurans which adversely affects human health and pose pollution problems when released into the environment. It was believed that, halogenated flame retardant based on either chlorine or bromine are most widely used, as the (–C–Cl–) or (–C–Br–) bonds are relatively weak and thermally labile. Whereas, fluorine containing compounds have very high thermal stability as (–C–F–) bond dissociation energy is very high and on contrary iodine based compounds are unstable at processing temperature of most of the polymers. Therefore by considering the environmental hazards associated with the utilization of halogenated flame retardant and the way of their pyrolysis mechanism, recently research has been focused on the development of halogen free flame retardants.[4–9]

In contrast to conventional thermally cured and solvent-borne resins, UV-curable coating systems have wide range of applications due to their outstanding features like free of volatile organic compounds (VOCs), being energy saving, ability to coat heat-sensitive devices and circuits, lower process cost, high chemical resistance, good chemical and solvent resistance, increase production capacity and broad range of applicable changes in formulation. Thus, photocured technologies perfectly fit today’s demands, as this technology overcomes the number of stringent environmental legislation. In recent time, the photocurable coating market is expanding at a rapid pace to wider applications areas. As a result, new requirements emerge for photocurable materials. As one of the most critical requirements, flame retardancy for photocurable coatings is highly desired.[10–16]

Photopolymerizable resins are being increasingly used in various applications, mainly in the coating industry, graphic arts and microelectronics, for replacing conventional thermally cured solvent-based coatings. However, most of these UV-curable resins have many superior properties like, excellent moisture, solvent and chemical resistance, toughness, superior mechanical properties and have good adhesion to many substrates. But they can catch fire in the presence of ignition source due to their carbonaceous backbone which involves not only the health risk but also lose their mechanical strength, thus limits their applications where flame retardant performance is required.[17,18] Therefore, considering the environmental hazards associated with use of halogenated flame retardants, recent research has focused on development of halogen free environmentally friendly flame retardant.

The current environmental legislation regarding the utilization and hazardous consequences of organo-halogen flame retardants, force polymer chemist to developed environmentally friendly flame retardant materials. Recently organo-boron compounds are considered as environmentally friendly and used extensively after organo-phosphorus compounds as flame retardant either solely or along with nitrogen and phosphorous based flame retardants for their synergistic effect to improve the flame retardant ability of the polymers. Organo-boron compounds can act as an effective flame retardant due their ability to form the impermeable glassy coating upon their degradation. The formed glassy coating onto the surface of polymeric materials, function like a protective barrier and fascilate the exclusion of oxygen and prevent the further propagation of combustion.[19] Upon thermal decomposition, boron containing flame retardants produce boron oxide in the condensed phase and alters the decomposition process of the polymer in favour of carbonaceous char rather than CO or CO_2_.[20]

The most widely used application of epoxy resin is the encapsulation of microchips in electronic devices (printed circuit board) and transportation (automobile and aircraft) where the resistance to fire is of prime importance. Thus to avoid fire hazards associated with the materials, the incorporation of polymers which can have inherent flame retardant properties is essential. Flame retardants can be incorporated in polymer backbone by two approaches; either by physical blending (additive approach) or by covalent bonding (reactive approach). In the case of additive approach, due to the loose chemical bonding, the incorporated flame retardant material can be leached out or migrate which in turn severely affects the flammability of materials. However in reactive approach, the flame retardant material was covalently bonded to the main polymer backbone which is responsible for the improvement in the flame retardant performance of the polymer and it is less likely get into the environment to cause damage to the ecosystem.

In this manuscript, the efforts have been taken to developed halogen free reactive flame retardant. Environmentally friendly boron based reactive flame retardant for UV-curable epoxy oligomer was successfully synthesized and characterized. The reactive flame retardant was utilized to formulate UV-curable wood coatings at different weight fractions i.e. 5–25 wt %. The effects of varying concentrations of boron acrylate reactive diluent (BARD) on thermal stability, flame retardancy, oligomer viscosity and coatings properties have been studied.

## 2. Experimental

### 2.1. Materials

Phenyl boronic acid (PBA, 99%), Glycidyl methacrylate (GMA, 99%) and Tetrahydrofuran (THF, 99%), Triethylamine (99%) and Sulphamic acid were purchased from S. D fine chemicals and used as received. Epoxy acrylate oligomer (DESMOLUX-2266) was provided by Bayer material. Photoinitiator (Irgacure184) was received from BASF Pvt. Ltd India.

### 2.2. Synthesis of BARD

**Figure 1.**
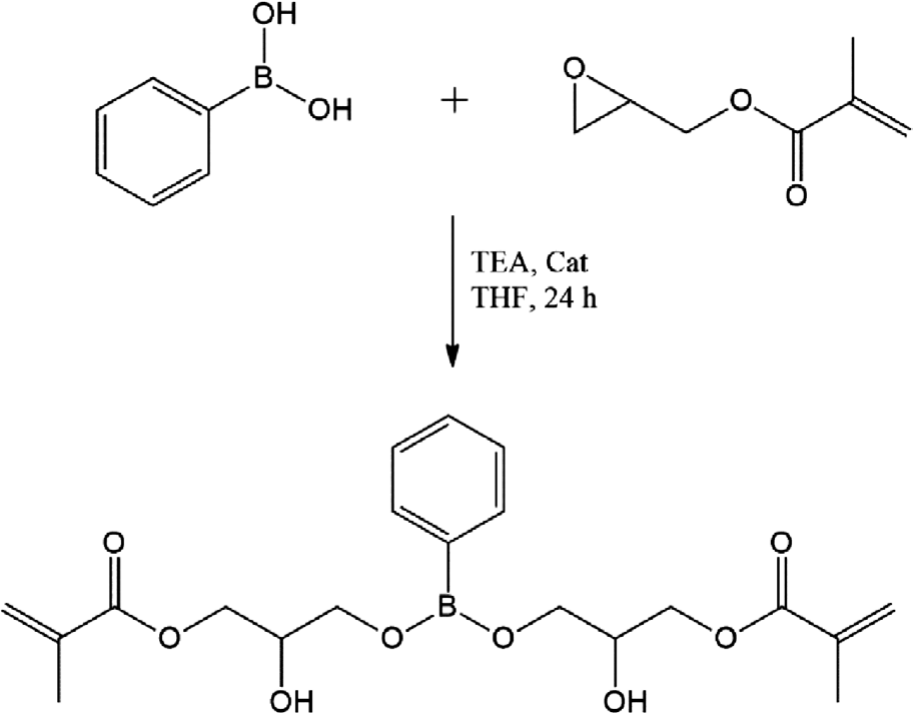
Synthesis of boron acrylate reactive diluent (BARD).

A 250 ml three-necked round bottom flask equipped with magnetic stirrer was charged with (0.1639 mol, 23.28 g) of GMA and sulphamic acid as a catalyst (0.5 mol %) in THF (50 ml), respectively. The mixture was then stirred gradually and the solution of PBA (0.0819 mol, 10 g) and triethylamine (1 mol %) in THF was added dropwise over a period of 1 h. After completion of addition, the reaction was continued for 24 h at room temperature. The progress of reaction was monitored by thin layered chromatography using hexane and ethyl acetate as an eluent (80:20). After completion of the reaction, the catalyst was filtered out and a solvent was removed under reduced pressure with the help of rotary evaporator. The yellowish liquid product was collected. The yield of product was 97% (Figure 1).The reaction scheme for synthesis of BARD is shown in Figure 1.

### 2.3. Preparation of UV-curable wood coatings

UV-curable formulations were prepared by mixing epoxy acrylate oligomer and boron based reactive diluent in various weight fractions .i.e. from 5 to 25% with constant photoinitiator concentration (3 wt %). Each formulation was prepared in a beaker with vigorous stirring. The prepared formulations were coated onto prepared wood panels (10 × 7 cm) using a bar applicator obtaining a uniform layer. Free films were prepared by pouring the viscous formulations onto the Teflon molds. Finally, the wet formulations were hardened after three passes by passing to a UV irradiation of a medium-pressure mercury lamp (365 nm) built into a UV machine. The speed of the belt is kept at 25 m/min (exposure time 20 s). The formulation for UV-curable wood coating is shown in Table 1.

**Table 1.** Formulation for UV-cured wood coating.

Ingredients (g)	Wt % of reactive diluent				
	5	10	15	20	25
BARD	5	10	15	20	25
Oligomer	92	87	82	77	72
Photoinitiator	3	3	3	3	3
Total	100	100	100	100	100

## 3. Characterization and measurements

### 3.1. Iodine value

Iodine value was determined according to the Wijs method by using iodine monochloride solution in acetic acid and carbon tetrachloride. The excess halogen was determined by adding KI solution and titration of liberated iodine with sodium thiosulphate solution (ASTM D1959-97).

### 3.2. Fourier-transform infrared spectroscopy

Fourier-transform infrared (FTIR) analysis was conducted on Shimadzu (8400 s, Japan) instrument using attenuated total reflection technique and the spectrum obtained in the wavelength range of 4000–600 cm^−1^.

### 3.3. Nuclear magnetic resonance spectroscopy

^1^H and ^11^B NMR analysis were conducted on 400 MHz using Bruker Biospin (Avance AV500WB, Germany) in CDCl_3_ with Tetramethylsilane as an internal standard. BF_3_·OEt_2_ used as an external standard (for ^11^B NMR).

### 3.4. Viscosity measurement

Viscosity measurement was performed at 30 °C using a rheometer (MCR, 101, Anton Paar, Graz Austria) in the cone and plate geometry with a diameter of 35 mm, over the shear rate ranges from 0.01 to 100 s^−1^.

### 3.5. Contact angle measurement

The contact angle measurement of UV-cured coatings were performed using standard goniometer (250, Rame-hart instrument company, USA) by applying sessile drop method.

### 3.6. Gel content measurements

Gel content measurements were determined by weighing a cured film samples accurately and extracted in methyl ethyl ketone (MEK) at room temperature for 24 h. The UV-curable films were dried in vacuum oven at 80 °C till to achieve a constant weight. Gel content of the UV-cured film was calculated by equation below:

$$\text{Gel content}\;\left(\%\right)=W_{\text{after}}/W_{\text{before}}\times 100$$

where *W*_after_ and *W*_before_ are the weight of cured coating films after extraction and before the extraction respectively.

### 3.7. Water absorption behaviour

Water absorption behaviour of UV-cured films was investigated according to ASTM D570. UV-cured films were dried at 80 °C in a vacuum oven until constant weight was achieved. Then, the films were immersed in water at room temperature for 24 h. UV-cured films were removed, patted and dry with a lint-free cloth, and weighed. The percent water gain was determined from the equation:

$$\text{Water absorption}\;\left(\%\right)=W_{\text{after}}-W_{\text{before}}/W_{\text{before}}\times 100$$

where *W*_before_ and *W*_after_ are the weight of cured coatings films before the exposure and after exposure to water absorption respectively.

## 4. Thermal analysis

### 4.1. Thermogravimetric analysis

Thermogravimetric analysis (TGA) of cured polymeric films was performed using TA SDT Q600 thermal analyzer at a scanning rate of 10 °C/min under N_2_ and air. About 10.0 mg of the UV-cured sample was put in an alumina crucible and heated from 30 to 600 °C

### 4.2. Differential scanning colorimetry

The glass transition temperature of cured coating films was determined using differential scanning calorimetry (DSC) TA Q100 analyzer (T.A. Instruments, USA).The sample was taken in an aluminium pan and was kept for thermal analysis under the nitrogen atmosphere at a heating rate of 10 °C/min in the temperature range of 0–100 °C.

### 4.3. The limiting oxygen index

The limiting oxygen index (LOI) is the minimum concentration of oxygen determined in flowing mixture of oxygen and nitrogen that will just support combustion of a material. The flammability and thermal behaviour of cured film were studied by LOI.

### 4.4. UL-94 vertical burning test

UL-94 vertical burning test was carried out according to ASTM D1356-2005. Six test sample with dimensions 127 × 12 × 2 mm was suspended vertically and inginated, by using LPG Bunsen burner. The end of the sample bar was ignited, ignition was carried out for 10 s.

## 5. Optical and mechanical properties of coatings

### 5.1. Gloss of UV-cured coatings

The gloss of UV-cured coatings was measured using Rhopoint gloss meter at 60°angle digital as per ASTM D523-99.

### 5.2. Hardness

Hardness is a measure of the resistance of coating to the mechanical forces, such as pressure, rubbing or scratching. The hardness of the UV-cured coating applied onto the wood panel was measured by pencil hardness test according to ASTM D 3363. Pencils of various degrees of hardness were drawn over the coating surface to determine which pencil causes an indentation.

### 5.3. Cross-cut adhesion test

Cross-cut adhesion test specifies a procedure for assessing the resistance of the coatings to separation from the substrate when a right angle lattice pattern was cut into the coating, penetrating through the substrate. The adhesion test was performed using cross-cut adhesion tester on the wood substrate according to ASTM D 3359.

### 5.4. Solvent resistance

Solvent resistance of UV-cured coating was determined by ASTM D 5402-93 for assessing the solvent resistance of cured coating by rubbing the coating with a cloth saturated with the MEK.

### 5.5. Stain resistance

The stain resistance of coatings was determined according to ASTM D3023-98. Different stains were applied on coatings and covered with filter paper for 24 h; then the panels were washed with water and subsequently with ethanol.

## 6. Results and discussion

### 6.1. Determination of the iodine value

The iodine value of synthesized BARD was estimated by Wijs method and found be 115.8 (g of *I*_2_/100 g of resin). The theoretical iodine value for BARD is 125.04 (g of *I*_2_/100 g of resin).

## 7. Characterization

### 7.1. FTIR analysis of BARD

**Figure 2.**
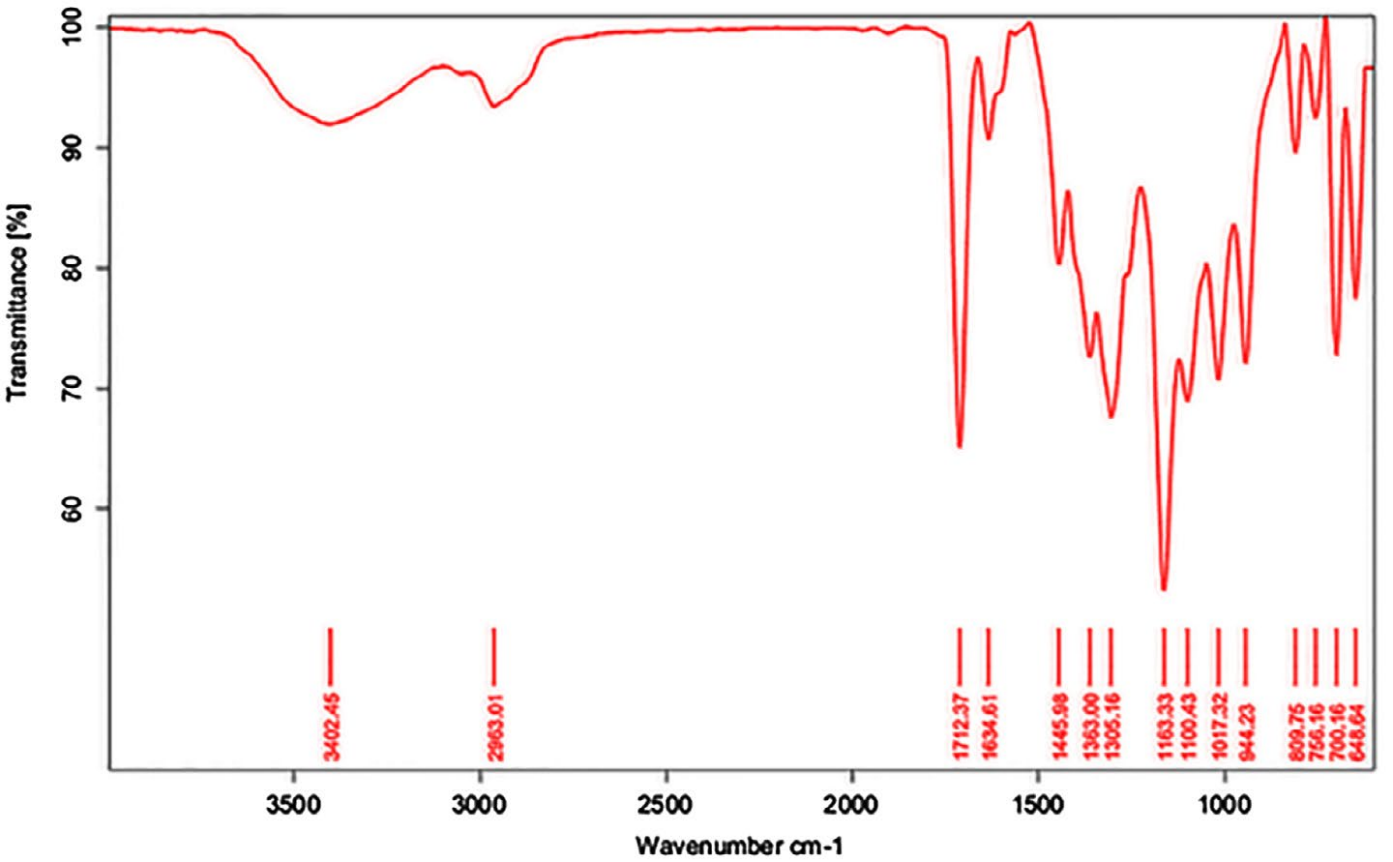
FTIR analysis of BARD.

FTIR analysis of synthesized boron diacrylate reactive diluent shown in Figure 2. The characteristic absorption at 1712.37 cm^−1^ corresponds to the carbonyl group (–C=O), the presence of peak at 2963.01 cm^−1^ assign to (–CH_2_) aliphatic stretching, the typical broad peak at 3402.45 cm^−1^ corresponds to the hydroxyl functionality (–OH) and absence of peak at 842 cm^−1^ (oxirane stretching) indicates that, the ring opening reaction of PBA with GMA was taken place successfully. The peak at 1163.33 corresponds to (–C–O–) absorption, the peaks at 1305.16 and 648.64 cm^−1^ corresponds to the (–B–O–C) and (–B–C–) stretching respectively, the presence of peaks at 1634.61 and 1445.98 cm^−1^ corresponds to aliphatic unsaturation and aromatic nucleus from PBA respectively.

### 7.2. ^11^B NMR analysis

**Figure 3.**
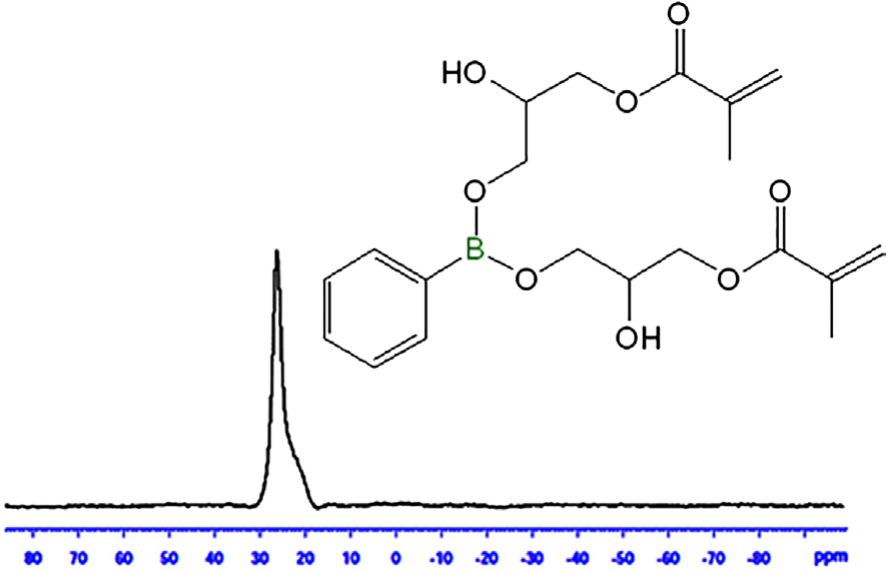
^11^B NMR of boron BARD.

^11^B NMR analysis of BARD depicted in Figure 3.The characteristic singlet at 26.4 ppm corresponds to the alkyl boronates absorption (–C–B–O– linkage) which generally resonates at very down field region in NMR spectrum.

### 7.3. ^1^H NMR analysis BARD

**Figure 4.**
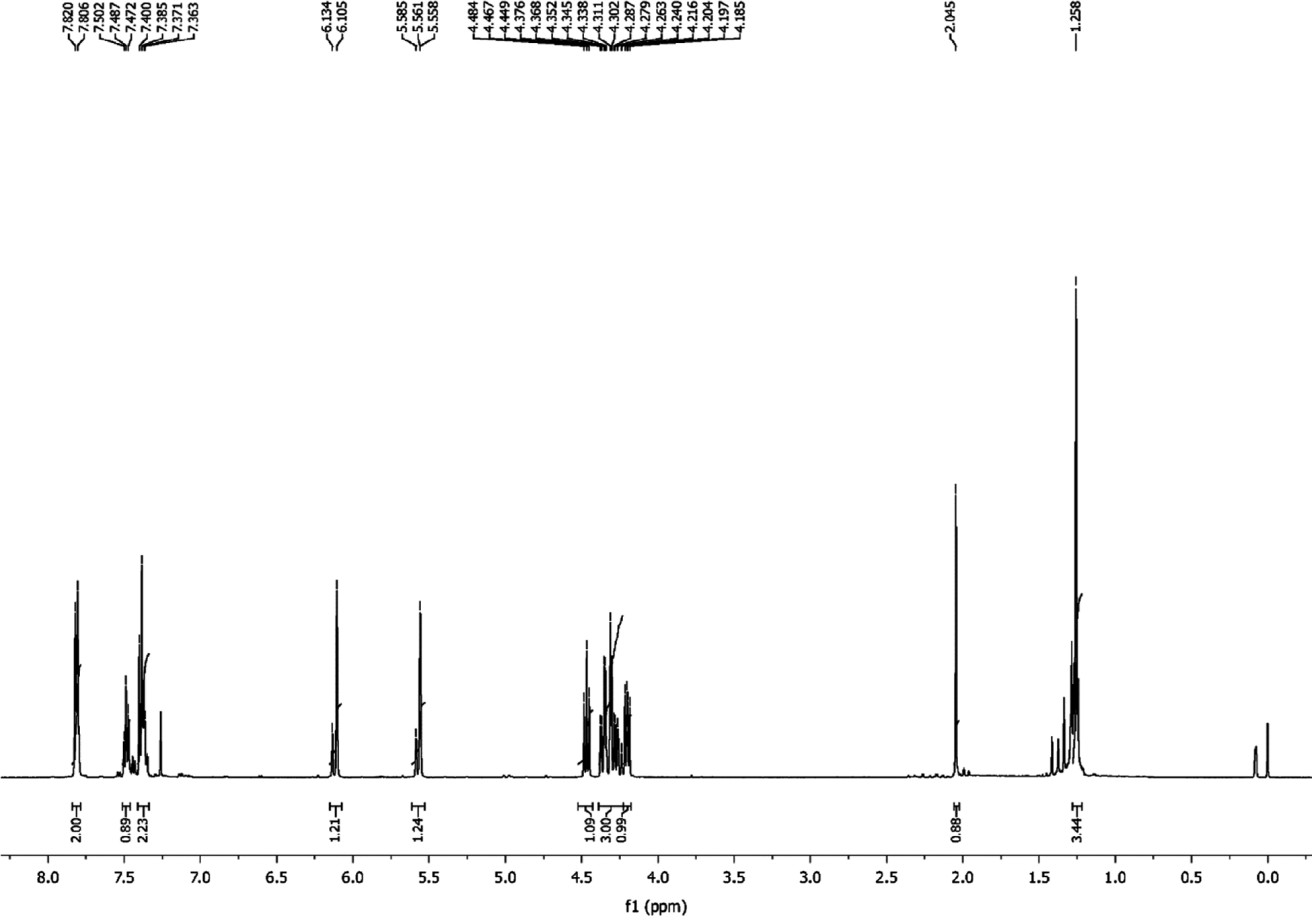
^1^H NMR of BARD.

Figure 4 shows the ^1^H NMR for synthesized boron di-acrylate. The chemical shift at 7.82–7.8 ppm corresponds to the aromatic region of the boron di-acrylate. The olefinic protons from acrylate moiety appear as doublet at 6.13 and 6.10 ppm, respectively. The protons flank between the carbonyl and hydroxyl group appear at 4.84 ppm whereas the protons which are between the electronegative oxygen atom and the carbon to which hydroxyl group is attached appear at 4.37 ppm. The protons (–CH–OH) appear at 4.28 ppm. The singlet at 2.04 ppm corresponds to the proton of hydroxyl group. The proton of alkyl groups of acrylates appear at 1.25 ppm. The solvent peak appear at 7.25 ppm.

### 7.4. Viscosity measurement

**Figure 5.**
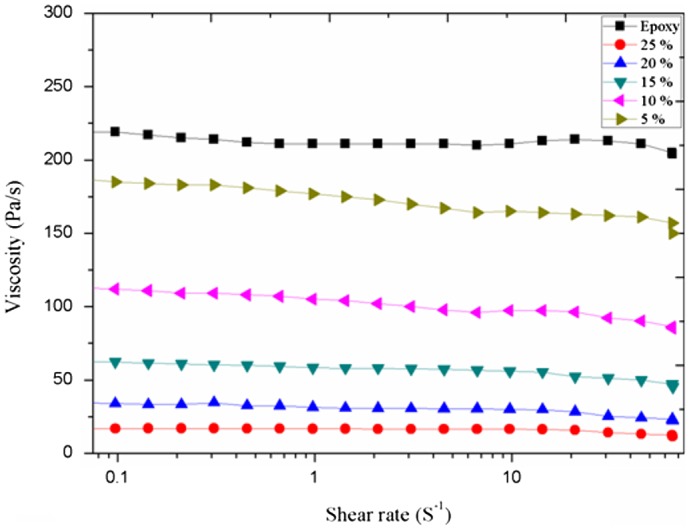
Effect of reactive diluent concentration on the viscosity of oligomer.

To reduce the viscosity of coating formulation and make the application effortless are the prime functions of reactive diluent. Therefore, an effect of BARD concentrations on the viscosity of coating formulations has been studied and shown in Figure 5. It is clearly seen, that the viscosity of epoxy acrylate oligomer is very high but with increasing concentration BARD in coating formulations, the viscosity of the epoxy acrylate oligomer effectively decrease, being the extent was highest for the formulation which contains 25% weight fraction of BARD. These results clearly indicate the effectiveness of reactive diluent in lowering the viscosity of coating formulation.

### 7.5. Contact angle analysis

**Figure 6.**
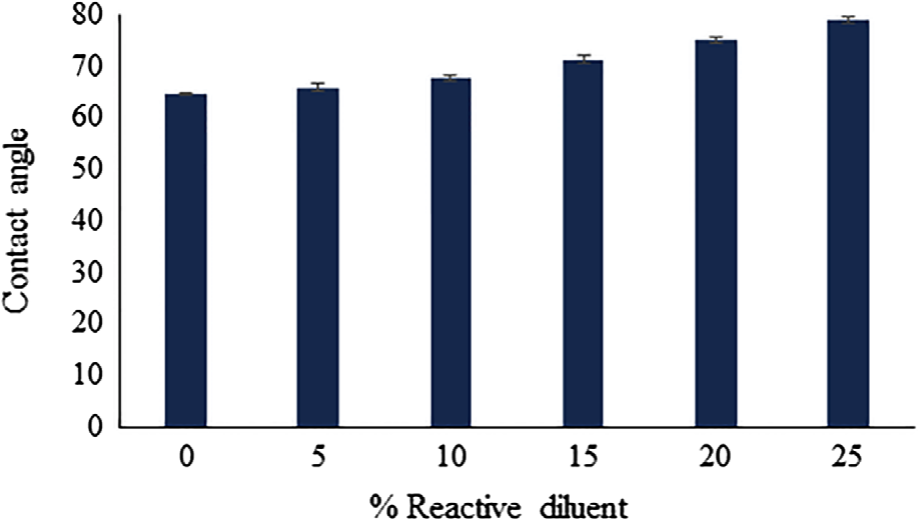
Contact angle analysis of UV-cured coatings.

In order to investigate the surface characteristics of UV-cured wood coatings, water contact angle measurement was performed. It was observed that, upon increasing the concentration of BARD in the coating formulation, the number of unsaturations available for photo-polymerization with epoxy acrylate oligomer was increased, as a consequence, the extent of photo-polymerization was rise abruptly. The boost in photo-polymerization leads to the formation of cross-linked three-dimensional interpenetrating networks which makes the coating surface hydrophobic in nature. Thus, it can be concluded that, the contact angle has linear relationship with increased concentrations of BARD (Figure 6). Contact angle analysis of cured coatings is shown in Figure 6.

### 7.6. Gel content measurement

**Figure 7.**
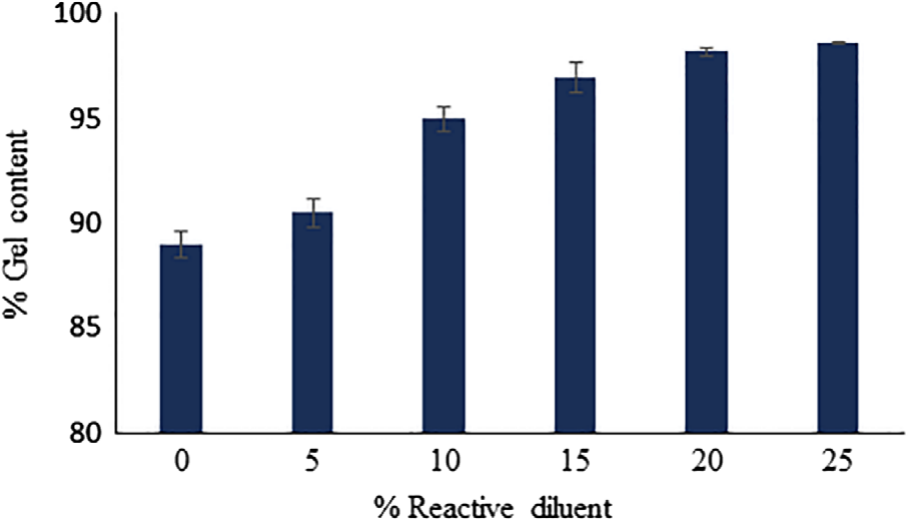
Gel content of UV-cured coatings.

The extent of curing of the photo-polymerized coating films containing varying concentrations of BARD is shown in Figure 7. The degree of photo-polymerization of epoxy acrylate oligomer is a function of varying concentrations of BARD i.e., the degree of unsaturation upon exposure to UV irradiation, and was determined by solvent extraction in terms of gel content. Gel content is a measure of curing and crosslinking of the coatings. It was observed, that the gel content increased linearly with increasing weight fraction of BARD in coating formulation, which also leads to increased extent of photo polymerization. Increased concentrations of BARD in coating formulation not only reduced the viscosity of the oligomer but also enhance the diffusion of photoinitiator radical, being responsible for a greater extent of photo-curing which leads to the formation compact three-dimensional networks i.e. crosslinking. This explanation is well supported by the viscosity measurement of analysis.

### 7.7. Water absorption behaviour of UV-cured coatings

**Figure 8.**
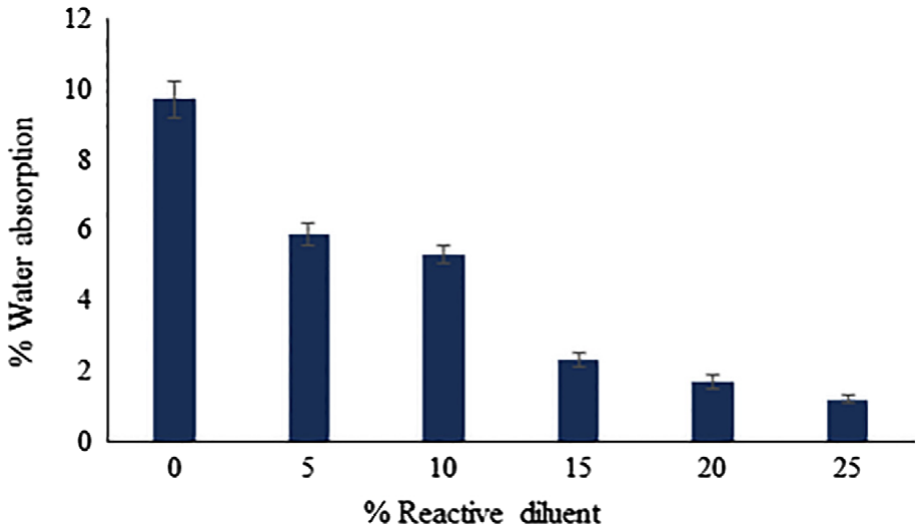
Water absorption behaviour of UV-cured coatings.

Generally, absorbed water has a devastating effect on coatings properties, thus water absorption behaviour of coatings is of great interest to determine their end used application. The water absorption behaviour of cured coatings is shown in Figure 8. It was found that, as the concentration of BARD in UV-curing composition was increased, the photo-polymerization of epoxy acrylate oligomer led to the formation of more compact interpenetrating network upon UV exposure. This network led to a decline in the presence of free volume or space in coating thereby avoiding entry of water molecules. Moreover, the epoxy acrylate oligomer backbone includes an aromatic nucleus which is hydrophobic in nature, hence helping to improve the water resistance of cross-linked polymeric coating and thus retarding its water absorption capacity.

## 8. Thermal analysis

### 8.1. Thermogravimetric analysis

**Figure 9.**
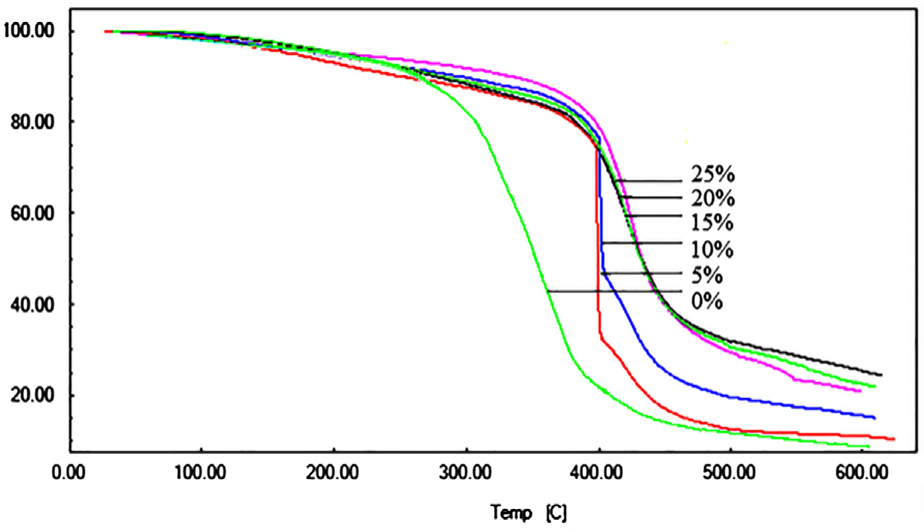
Thermogravimetric analysis of UV-cured films.

TGA is one of the most widely used technique for rapid evaluation of thermal stability and the decomposition behaviour of polymers. The thermal stability of a polymeric material is very important when used as flame retardant which mainly concerns with the release of decomposition products and the formation of residual char. The thermal stabilities of the UV-cured thermosets with a various weight fraction of BARD were evaluated by TGA with a temperature range from the 30 to 600 °C in nitrogen atmosphere and depicted in Figure 9, shows the behaviours of the thermal degradation for these thermosets, and the obtained data are summarized in Table 2. TGA thermogram shows two steps degradation, wherein the first thermal degradation occurs due to the decomposition of soluble part or uncross-linked labile segments in polymer backbone while the second degradation is related to the cross-linked three-dimensional networks.

**Table 2.** Thermogravimetric analysis of UV-cured films along with char yield.

Sr. no	Wt % of BARD	Onset (*T* °C)	End set (*T* °C)	% Char yield
1	5	412	400	7
2	10	404	409	17
3	15	397	419	21
4	20	387	430	24
5	25	371	442	28

It was observed that, upon increasing the concentrations boron containing compounds in polymers, possess higher char yield due to the flame retardant action of boron.[21] Organoboron reactive flame retardant is well known for their effective flammability and which was originated by the formation of a non-penetrable surface layer of boron oxide during degradation of cross-linked polymeric material.[22] It was believed that, during the process of combustion, boronic acid losses water molecule and convert primarily in to metaboric acid, further it transform into the HBO_2_ and finally to the boron oxide (B_2_O_3_). Boron oxide was formed during the degradation process from the cleavage of (B–C) and (B–O–C) bonds via pyrolysis, which led to formed glassy surface layer which not only provides thermal insulation but also acts as a protective barrier against fuel transfer and reduces thermal conductivity which primarily resist the heat transfer from flame to polymer thus preventing the melt, flow and further thermal decomposition of the material.[23–26] Therefore, helping in improving the flammability of the material.

### 8.2. Differential scanning calorimetry

**Figure 10.**
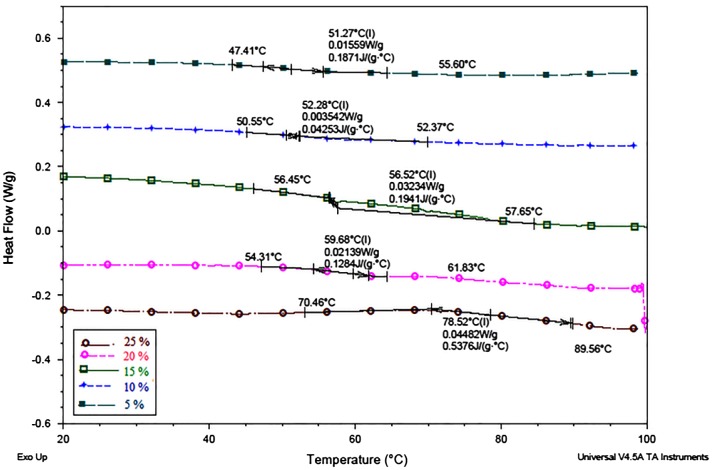
Glass transition temperature of UV-cured coatings films.

The effects of different concentrations of BARD on the glass transition temperature of photo cured coating films were evaluated by mean of DSC and shown in Figure 10. It was noticed that, as there is a raise concentration of BARD in UV curing formulation, the *T*_g_ of cured coatings films were found to be increased. The coatings with different concentration of BARD in coatings i.e. 5–25 wt %, the *T*_g_ was shift from 51 to 78 °C, respectively. The increase in glass transition temperature of coating films is the outcome of a boost in the extent of photo-polymerization upon UV exposure which leads to increase the cross-linking density of cured films. Thus, raise in glass transition temperature of UV-cured films is a function of increased weight fraction of BARD and extent of photo-polymerization. The rigid aromatic backbone of epoxy acrylate oligomer is also responsible for the rise in glass transition temperature of UV-cured coating films.

### 8.3. LOI analysis

**Figure 11.**
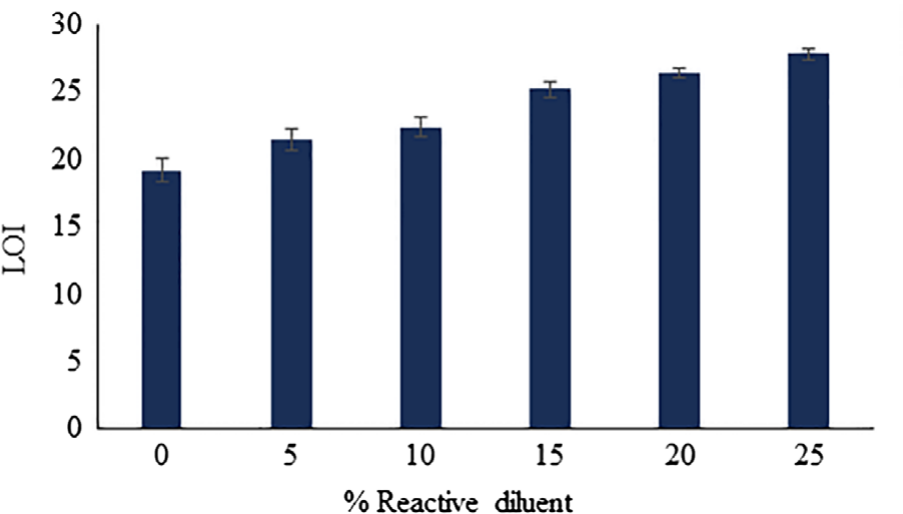
LOI analysis of UV-cured coatings.

The effectiveness of flame retardant properties of BARD based UV-cured films were assessed by means of LOI measurement. LOI measures the ease of extinction of materials as the minimum concentration of oxygen that will just sustain combustion in a candle like the configuration of a top-ignited vertical test specimen and depicted in Figure 11. It was reported that, higher the content organoboron compound in UV-cured composition, there is significance rise in LOI was observed.[27,28] The raise in LOI shows linear a relationship with the increased in boron content. LOI revealed that, as the content of incorporated BARD in coating formulation increases, the resistance of UV-cured coating films towards fire become enhanced. The enlarge covalently bonded boron content is responsible for the generation of more amount of born oxide which formed upon the pyrolysis of (B–C) and (B–O–C) bonds during the process of combustion which helps in the formation of more amounts of residual char which act as a protective barrier against the fire. The residual char formation increases the decomposition temperature of epoxy oligomer by preventing the heat transfer from source to the polymeric material. This conclusion is in accordance with char yield data obtained from TGA, indicating that increasing contents of boron and aromatic backbone in the epoxy acrylate oligomer would help to improve the flame-retardant assets of UV-cured coatings.

### 8.4. UL-94 vertical burning test

UL-94 vertical burning test was carried out to investigate the burning behaviour of UV-cured boron based thermoset coatings. All UV-cured coatings with the different fractions of BARD achieved UL-94V-0 classification in flammability test. All the UV-cured coating with different weight fraction of diluent quenched the fire within a period of 5–10 s when the flame was removed during the test and demonstrated the self-extinguishable behaviour. There was no dripping char residue after completion of burning test, indicating the good structural stability of residual char which is the consequence of increase boron content and molecular structure of epoxy acrylate oligomer.

## 9. Optical and mechanical properties of coatings

### 9.1. Gloss of UV-cured coatings

**Figure 12.**
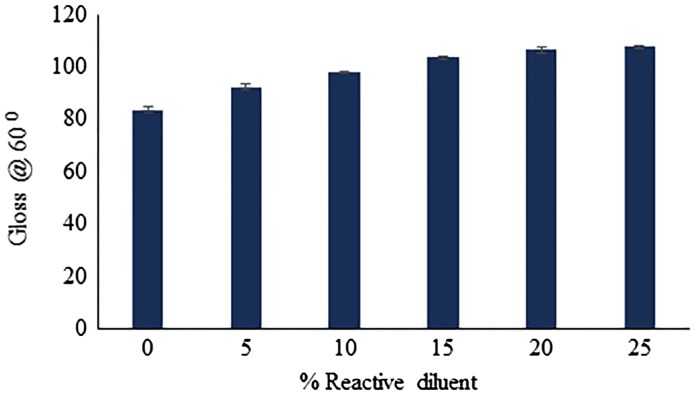
Gloss of UV-cured coatings.

The gloss of the coating is related to the ability of material to flow and level, and its surface smoothness. The gloss of UV-cured coatings with varying weight fractions of BARD is depicted in Figure 12. As the concentration of BARD in the coating formulation was increased, the gloss also tended to rise. This can be attributed due to the fact that, increased in BARD concentrations results in the greater extent of photo-polymerization and degree of crosslinking, which led to improving the surface smoothness of the coatings. Increased concentration of BARD also leads to reducing the viscosity of oligomer, as a result the flow and levelling also improved.

### 9.2. Stain resistance of coatings

Stain resistance of coating is a vital criterion for the protecting wood from liquid spills and stains. Stain resistance of UV-cured wood the coatings was determined according to ASTM D3023-98 using permanent ink, ballpoint ink, turmeric, lipstick, shoe polish and tincture of iodine, shown in Table 3. The coatings showed excellent stain resistance due to the formation of interpenetrating network upon UV exposure. It was observed that, as the concentration of reactive diluent in coating formulation increases, the crosslinking density rises very abruptly which results in the formation more compact the three-dimensional interpenetrating network which assists in reducing the free volume or void on coating surface which help to improve the stain resistance of coatings.

**Table 3.** Stain resistance of UV-cured wood coatings.

Wt % of BARD	Iodine tincture	Turmeric	Shoe polish	Permanent ink	Ball-point ink	Lip stick
5	3	3	5	3	5	5
10	4	4	5	5	5	5
15	5	5	5	5	5	5
20	5	5	5	5	5	5
25	5	5	5	5	5	5

### 9.3. Mechanical properties of coatings

The mechanical properties of UV-curable coatings were influenced by the chemical composition of the resin, weight fractions of reactive diluent and concentration of photoinitiator. After completion of the curing process, several properties of coatings properties like solvent resistance, cross cut tape adhesion and pencil hardness were evaluated according to the corresponding ASTM standards and summarized in Table 4. The UV-cured coatings exhibited excellent chemical resistance indicated by MEK double rub cycles. The solvent double rub test was done up to 500 cycles. It was observed that, there is no deterioration in the coatings such as loss of gloss or dissolution of coating films. The cross-cut tape adhesion of coating was found to decrease as the concentrations of BARD in the formulation increased. This was attributed to the fact that, as the concentration of BARD in the formulation was increased, the extent of photo-polymerization increased, leading to the formation of more crosslinked network, resulting in brittle coating surface. The pencil hardness of coatings was found to decrease on increasing the concentration of BARD.

**Table 4.** Coatings properties.

Wt % of BARD	Solvent resistance	Pencil hardness	Adhesion
5	>450	6H	5B
10	>450	6H	4B
15	>450	6H	4B
20	>450	5H	3B
25	>450	4H	3B

## 10. Conclusions

Environmentally friendly BARD was successfully synthesized and incorporated into UV-curable wood coatings system along with epoxy oligomer in different weight fractions. The UV-cured coating films were evaluated for their flame retardant performance by means of TGA, LOI and UL-94 burning test, the films shows admirable flame retardancy. It was also revealed that, optical and mechanical properties of the coating like gloss, solvent resistance, cross-cut adhesion and pencil hardness were also affected by the concentration of BARD. In addition, contact angle, gel content and water absorption behaviour of coating were also studied. Here, the effluence of varying concentration of BARD on coating properties, gel content, contact angle and water absorption behaviour is the outcome of crosslinking density builds during the curing process. The ability of reactive diluent to cure rapidly under UV light is also beneficial economically and minimizes any practical emission of VOCs.

## Disclosure statement

No potential conflict of interest was reported by the authors.

## Funding

This study is financially supported by the University Grants Commission, New Delhi, India.
